# Vienna Letter

**Published:** 1881-08

**Authors:** 


### ARTICLE XII.

VIENNA, June 16.

EDITORS CHICAGO MEDICAL JOURNAL AND EXAMINER :—The famous operation, the excision of the cancerous pylorus, has scarcely realized the bright anticipations of its creator, perhaps because the cases hitherto operated upon have not been judiciously selected. Some nine stomach resections have been already reported, but one of the patients still surviving. Even the first, Billroth's own, operated January 29, has recently (about May 26), succumbed to a recurrence of the disease. Besides these there have been doubtless several cases *not* reported, as I know personally in one instance which occurred in Vienna. It is of course to be expected that patients will wait long, usually too long, before submitting themselves to an operation so formidable. Yet in the Continental hospitals, where patients place implicit trust in the surgeon, opportunities will doubtless occur for a fair and thorough test of the value of this daring but after all rational innovation. The one case which has not yet been terminated fatally, was that of Billroth's assistant, Woelfler, performed in April last, in which, according to the operator's statement, the malignant disease was confined absolutely to the stomach, all other organs, including the lymphatic glands, being apparently unaffected. As may be imagined, this patient will be watched with anxious interest by all friends of the operation. Woelfler has recently published a monograph, with plates, describing the operation and modifications as performed by Billroth, published by Braumueller & Co., Vienna.

Another novelty, less startling, but perhaps more valuable, has been within the last few months introduced and pretty thoroughly tested in Vienna; the local application of iodoform to unhealthy, particularly tuberculous, granulations. Brought out by Mosetig, it was soon used quite extensively in Billroth's wards, and with the most satisfactory results, according to the report of B.'s assistant, Mikuliez, at the surgical congress in Berlin. All indolent, granulating surfaces, but particularly the fungous granulations so often observed after chronic synovitis with ulceration of cartilage, have been observed to assume rapidly a healthy appearance, and exhibit an unusually favorable course after a few daily applications of iodoform crystals, so that this procedure has taken a recognized place in the armament of the Vienna surgeon.

By no means the least important of recent Vienna promulgations, are the researches of Dr. Jos. Gruenfeld, with the endoscope, or rather urethroscope. Ever since Désormeaux's presentation of his complicated endoscope in Paris, 1867, this branch of investigation has received more or less fitful and transitory attention from various specialists. Nearly all have contented themselves with the construction of instruments involving abstruse optical principles, culminating in the recently constructed, very beautiful, elaborate and expensive, but (for the urethra) comparatively worthless electrical endoscope of Nitze in Dresden, made by Leiter, in Vienna. Meanwhile, Dr. Gruenfeld, with very simple instruments, was training the *eye*. Beginning in 1871, as assistant in Sigmund's extensive syphilis division of the Vienna General Hospital, he has steadily and perseveringly pursued the study, and has finally, during the last year or two, by means not simply of various publications on the subject, but also (and this is far more satisfactory) through many admirable demonstrations in the Royal Society of Physicians, earned deserved attention and recognition, not simply for himself and his method, but also for the subject—*urethroscopy.* This recognition took tangible shape in the form of an invitation by Billroth to write a volume for the " System of German Surgery" edited by Billroth and Luecke of Strassburg, now appearing. The book appeared last year, is being already translated into Italian, and will soon appear in English. That the author has somewhat exalted notions as to the future of endoscopy, is not unnatural ; but that he gives valuable information as to the investigation and treatment of pathological conditions in that small but troublesome field, the urethra, is acknowledged even by his fiercest opponents and critics. I was so fortunate as to enjoy for several months Dr. G.'s private instruction, and propose in future letters to give the JOURNAL readers the benefit of my experience.

Another interesting and perhaps practically valuable work is the investigation of the origin and formation of vesical calculi, by the young but already well-known specialist, Dr. Robt. Ultzmann, of Vienna. The work has not yet been published, but will be presented to the International Congress, in London, in August, illustrated by numerous micro-photographs of sections through vesical stones. This work has especial value from the fact that the rapidly increasing popularity of lithotripsy à la Bigelow, is fast removing the possibility of convenient work in this line of investigation.

---

THE next meeting of the American Dermatological Association will be held at Newport, R. I., on the 30th and 31st of August and the 1st of September next. Papers are expected from many of the members, including Drs. White and Wigglesworth, of Boston; Duhring and Atkinson, of Philadelphia; Heitzman and Sherwell, of New York ; Atkinson, of Baltimore; Hardaway, of St. Louis, and others.

